# Randomized trial of conventional versus radiofrequency needle transseptal puncture for cryoballoon ablation: the CRYO-LATS trial

**DOI:** 10.1007/s10840-022-01277-y

**Published:** 2022-06-24

**Authors:** Jason G. Andrade, Laurent Macle, Matthew T. Bennett, Nathaniel M. Hawkins, Vidal Essebag, Jean Champagne, Jean-Francois Roux, Bhavanesh Makanjee, Anthony Tang, Allan Skanes, Yaariv Khaykin, Carlos Morillo, Umjeet Jolly, Evan Lockwood, Guy Amit, Paul Angaran, John Sapp, Stephan Wardell, George A. Wells, Atul Verma, Marc W. Deyell

**Affiliations:** 1 University of British Columbia, Vancouver, Canada; 2 Center for Cardiovascular Innovation, 2775 Laurel St, Vancouver, BC V5Z 1M9 Canada; 3 Montreal Heart Institute, Université de Montréal, Montreal, Canada; 4 McGill University Health Centre, Montreal, Canada; 5 Hôpital Sacré-Coeur de Montréal, Montreal, Canada; 6 Université Laval, Quebec, Canada; 7 Université de Sherbrooke, Sherbrooke, Canada; 8 Rouge Valley Centenary Hospital, Ajax, Canada; 9 University of Western Ontario, London, Canada; 10 Southlake Regional Health Center, Newmarket, Canada; 11 Libin Cardiovascular Institute, University of Calgary, Calgary, Canada; 12 St. Mary’s Hospital, Kitchener, Canada; 13 Royal Alexandra Hospital, Edmonton, Canada; 14 McMaster University, Hamilton, Canada; 15 St. Michael’s Hospital, University of Toronto, Toronto, Canada; 16 Dalhousie University, Halifax, Canada; 17 University of Saskatchewan, Saskatoon, Canada; 18 University of Ottawa Heart Institute, Ottawa, Canada

**Keywords:** Transseptal puncture, Catheter ablation, Radiofrequency

## Abstract

**Background:**

Transseptal puncture to achieve left atrial access is necessary for many cardiac procedures, including atrial fibrillation ablation. More recently, there has been an increasing need for left atrial access using large caliber sheaths, which increases risk of perforation associated with the initial advancement into the left atrium. We compared the effectiveness of a radiofrequency needle-based transseptal system versus conventional needle for transseptal access.

**Methods:**

This prospective controlled trial randomized 161 patients with symptomatic paroxysmal atrial fibrillation undergoing cryoballoon pulmonary vein isolation to transseptal access with a commercially available transseptal system (radiofrequency needle plus stiff pigtail wire; RF + Pigtail group) versus conventional transseptal access (standard group). The primary outcome was time required for left atrial access. Secondary outcomes included failure of the assigned transseptal system, radiation exposure, and complications.

**Results:**

The median transseptal puncture time was significantly shorter using the radiofrequency needle plus stiff pigtail wire transseptal system compared with conventional transseptal (840 ± 323 vs. 956 ± 407 s, *P* = 0.0489). Compared to conventional transseptal puncture, fewer transseptal attempts were required (1.0 ± 0.5 RF applications vs. 1.3 ± 0.8 mechanical punctures, *P* = 0.0123) and the fluoroscopy time was significantly shorter (72.0 [IQR 48.0, 129.0] vs. 93.0 [IQR 60.0, 171.0] s, *P* = 0.0490) with the radiofrequency needle plus stiff pigtail wire transseptal system. Failure to achieve transseptal LA access with the assigned system was rarely observed (1.3% vs. 5.7%, *P* = 0.2192). There were no procedural complications observed with either system.

**Conclusions:**

The use of a radiofrequency needle plus stiff pigtail wire resulted in shorter time to left atrial access and reduced fluoroscopy time compared to left atrial access using conventional transseptal equipment.

**Trial registration:**

ClinicalTrials.gov identifier NCT03199703.

## Introduction

Atrial fibrillation (AF) is the most common sustained arrhythmia seen in clinical practice, being estimated to affect up to 3% of the overall population [1]. For many highly symptomatic patients, catheter ablation is an efficacious option, proven to significantly improve freedom from arrhythmia recurrence, produce clinically meaningful improvements in patient-reported outcomes (symptoms and quality of life), and significantly reduce healthcare resource utilization [2].

While transseptal puncture is required to achieve left atrial access for all AF ablation procedures, the use of the Arctic Front cryoballoon system (Medtronic, Minneapolis) for pulmonary vein isolation presents several unique considerations. Firstly, there is usually only a single transseptal puncture performed, which decreases the risk of complication associated with subsequent punctures. Second, the steerable guiding sheath required for cryoballoon ablation is of larger caliber than those used for conventional RF ablation procedures (15 French vs. 9.5–11 French), which may increase risk of perforation associated with the initial advancement into the left atrium. Third, a transseptal position low and anterior in the fossa is more technically desirable for cryoballoon ablation procedures due to difficulty engaging the right inferior pulmonary vein with the deflectable sheath.

Beyond AF ablation, the increasing number of procedures employing large bore sheaths and complex devices have led to advancements in transseptal technique and equipment. Two such technologies are the blunt-tipped radiofrequency needle, which was developed to increase precision and minimize the risk of perforation, and the atraumatic stiff body pigtail wire, which was developed to prevent left atrial perforation due to overshoot of the large caliber sheath as it passes through the interatrial septum.

We conducted a randomized trial to evaluate the effectiveness and safety of a radiofrequency needle transseptal system (TorFlex™ Transseptal Guiding Sheath, NRG® Transseptal Needle, ProTrack™ Pigtail Wire, Baylis, Montreal, Quebec, Canada) compared with a conventional transseptal system (conventional sheath, mechanical needle, and standard guidewire) for transseptal LA access during cryoballoon ablation procedures.

## Methods

### Study design

We conducted an investigator-initiated multicenter, parallel-group, randomized clinical trial at 14 centers in Canada. The trial design and conduct were overseen by a steering committee. The study protocol was approved by the institutional review committee at each center.

Data monitoring, collection, and primary data analysis were performed by the Cardiovascular Research Methods Centre (University of Ottawa) and the steering committee. The principal investigator prepared the manuscript. The authors vouch for the accuracy and completeness of the data and for the fidelity of this report to the trial protocol.

The study was funded by a peer-reviewed grant from the Cardiac Arrhythmia Network of Canada [grant number SRG-15-P15-001] with additional unrestricted financial support from Baylis Medical. The funding sources were not involved in study design; selection or monitoring of the participating centers; selection or enrollment of patients; data collection, storage, or analysis; data interpretation; manuscript preparation; nor in the decision to submit the manuscript for publication.

### Study participants

We enrolled adults (> 18 years) with symptomatic, treatment-naïve AF undergoing cryoballoon-based pulmonary vein isolation. All patients provided written informed consent.

### Randomization and study procedures

Patients were randomly assigned in a 1:1 ratio to transseptal puncture using the radiofrequency needle-based transseptal system or standard transseptal puncture. Randomization was performed with concealed allocation using permuted blocks according to a computer-generated allocation sequence with block sizes of 4 and 8 and stratified by center using web-based software.

Patients randomized to the radiofrequency needle-based transseptal system (**RF + Pigtail group**) underwent transseptal puncture with a large, preformed 18-gauge NRG RF transseptal needle through an 8.5-French TorFlex sheath, with the sheath curve selected at the discretion of the operating physician.

Patients randomized to the conventional transseptal group (**standard group**) underwent transseptal puncture with a large, preformed curve 18-gauge BRK needle (BRK or BRK-1 needle, Abbott) or alternate mechanical needle (SafeSept [Pressure Products, Inc.], HeartSpan [Biosense-Webster], or Cook Medical) through any 8.0–8.5-French non-Baylis sheath (FastCath [Abbott], or Preface [Biosense-Webster]) selected at the discretion of the operating physician.

#### Sheath preparation

Prior to use, the transseptal needle, sheath, and FlexCath catheter were flushed with heparinized saline. The assigned transseptal needle was then placed through the dilator and assigned transseptal sheath until the tip of the needle was visualized distally.

#### Transseptal puncture

Transseptal punctures were performed via right femoral venous access. Following access, the transseptal sheath and dilator were advanced to the superior vena cava over a guidewire under fluoroscopic visualization. Following guidewire removal, the contents of the dilator were evacuated and flushed with heparinized saline. The transseptal needle was flushed with heparinized saline, inserted into the dilator and sheath, and advanced under fluoroscopic guidance until the needle tip was located 2 to 5 mm proximal to the dilator tip. The needle, dilator, and sheath were pulled down as a unit until they were confirmed in the correct position in the fossa ovalis using fluoroscopy (± contrast injection), transesophageal echocardiography, or intracardiac echocardiography. While holding the dilator and sheath still, the needle tip was advanced out of the dilator. For the **RF + Pigtail group** RF energy was applied via the dedicated generator for 2 s, with repeated applications as needed until LA access was obtained. For the **standard group**, mechanical puncture was performed at operator discretion. Cross-over to an alternate needle or sheath type was allowed only if transseptal access was unsuccessful after multiple attempts, and further attempts to achieve LA access were deemed to be either futile or unsafe per the discretion of the operator.

#### FlexCath placement

The assigned guidewire (0.032″ in **standard group,** or ProTrack guidewire in the **RF + Pigtail group**) was advanced to the left superior pulmonary vein following confirmation of LA access (contrast media visualization in the LA under fluoroscopy, microbubbles observed in the LA with echocardiographic imaging, and/or left atrial pressure tracing). Once the guidewire was in place, the initial transseptal sheath was removed, and the FlexCath sheath was advanced over the wire into the left atrium and flushed as per standard procedure. Following FlexCath placement, the cryoballoon was prepped and inserted as per standard procedure. After the transseptal puncture was completed, all patients underwent a standard cryoballoon-based pulmonary vein isolation with the Arctic Front Advance (Medtronic) using standard techniques [3–5].

### Study outcomes

The primary outcome was the total time required for transseptal LA access, defined as time from the first pull-down of the needle/sheath/dilator apparatus from the superior vena cava to first entrance of the cryoballoon catheter into the left atrium. Secondary outcomes measures included the time from first pull-down to first entrance of the transseptal sheath into the left atrium, the time from engagement of the interatrial septum to FlexCath advancement into the left atrium, fluoroscopy time required for transseptal LA access, number of repositioning attempts, transseptal location, procedure success (defined as the ability of the assigned transseptal system to achieve left atrial access), and any procedural complication related to transseptal puncture (including but not limited to death, aortic puncture, pericardial effusion, cardiac tamponade, and stroke or systemic thromboembolism).

### Statistical analyses

For continuous variables, descriptive statistics were presented using mean ± standard deviation or median with interquartile range (IQR). For categorical variables, number and percentages were presented. Continuous variables were compared using t-test or the Mann–Whitney test. For categorical variables, Pearson’s Chi-square test or Fisher’s exact test were used. Variance was compared using the *F* test. Analyses of the primary and secondary endpoints were based on the intention-to-treat principle. All tests were conducted at an alpha level of 0.05. Analyses were performed using Prism 8 (GraphPad Software; San Diego, CA) and Stata 15 (StataCorp, College Station, TX).

## Results

A total of 161 patients were enrolled between January 2017 and December 2018 and randomized to either the RF needle transseptal system group (**RF + Pigtail group**, 74 patients) or conventional transseptal group (**standard group**, 87 patients). Baseline characteristics were balanced between groups (Table 1). The equipment used for transseptal procedure is presented in Table 2.

**Table 1:** Baseline characteristics

	RF + Pigtail	Standard	*P *value
*N*	74	87	
Age, mean (SD)	58.4 (11.5)	59.0 (11.1)	0.75
Sex	19 (26%)	28 (32%)	0.37
HTN	25 (34%)	35 (40%)	0.40
CAD	6 (8%)	5 (6%)	0.55
HF	2 (3%)	1 (1%)	0.47
Stroke	2 (3%)	3 (3%)	0.79
CKD	0 (0%)	1 (1%)	0.35
DM	9 (12%)	7 (8%)	0.38
CCSSAF, mean (SD)	2.4 (.9)	2.5 (.9)	0.54
OAC	57 (77%)	71 (82%)	0.47
Wt, mean (SD)	95.3 (35.2)	90.9 (24.5)	0.35
Ht, mean (SD)	177.0 (9.4)	173.5 (15.3)	0.094
bmi, mean (SD)	30.2 (9.9)	31.4 (16.7)	0.60
LAV, mean (SD)	38.6 (16.1)	34.4 (14.8)	0.17
LVEF, mean (SD)	60.3 (7.1)	59.4 (6.9)	0.45
TR	22 (39%)	28 (40%)	0.87
MR	20 (35%)	24 (34%)	0.92
ICE	33 (45%)	37 (43%)	0.79
TEE	24 (32%)	23 (26%)	0.40

Legend: *BMI*, body mass index; *CKD*, chronic kidney disease; *Ht*, height, in cm; *ICE*, intracardiac echocardiography; *LAV*, left atrial volume; *LVEF*, left ventricular ejection fraction; *MR*, moderate-severe mitral regurgitation; *TEE*, transesophageal echocardiography; *TR*, moderate-severe tricuspid regurgitation; *Wt*, weight, in kilograms

**Table 2:** Equipment used during transseptal procedure

	RF + Pigtail group	Standard group
Sheath		
• Torflex 37	2	
• Torflex 45	59	
• Torflex 55	13	
• Swartz SL0		13
• Swartz SL1		56
• Preface		18
Needle*		
• NRG 98 cm c0	1	
• NRG 98 cm c1	1	
• NRG 71 cm c0	24	2
• NRG 71 cm c1	48	4
• Cook	1	6
• HeartSpan		2
• BRK		30
• BRK-1		45
• SafeSept		2

^*^Numbers are greater than group allotment due to use of multiple needles in cases of primary failure

### Transseptal procedure

**Fig. 1:**
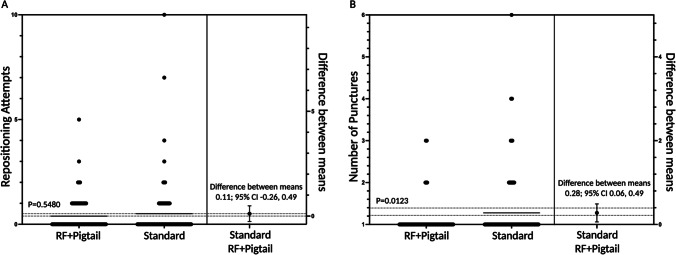
Repositioning attempts (**A**) and number of punctures (**B**) required for left atrial access

A mean of 1.39 ± 0.82 transseptal attempts (drop-down from superior vena cava to needle deployment) was required in the RF + Pigtail group, vs. 1.51 ± 1.44 in the standard group, *P* = 0.5480 (Fig. 1A). Following fossa engagement, a mean of 1.00 ± 0.50 RF applications was required for left atrial access in the RF + Pigtail group vs. 1.28 ± 0.82 mechanical needle puncture deployments in the standard group, *P* = 0.0123 (Fig. 1B). There was no significant difference in transseptal procedural success with the randomized equipment (98.7% in RF + Pigtail group vs. 94.3% in the standard group, *P* = 0.2192), with 1.3% of the RF + Pigtail group and 5.7% of the standard group crossing over to the alternate transseptal needle.

### Transseptal time

**Fig. 2:**
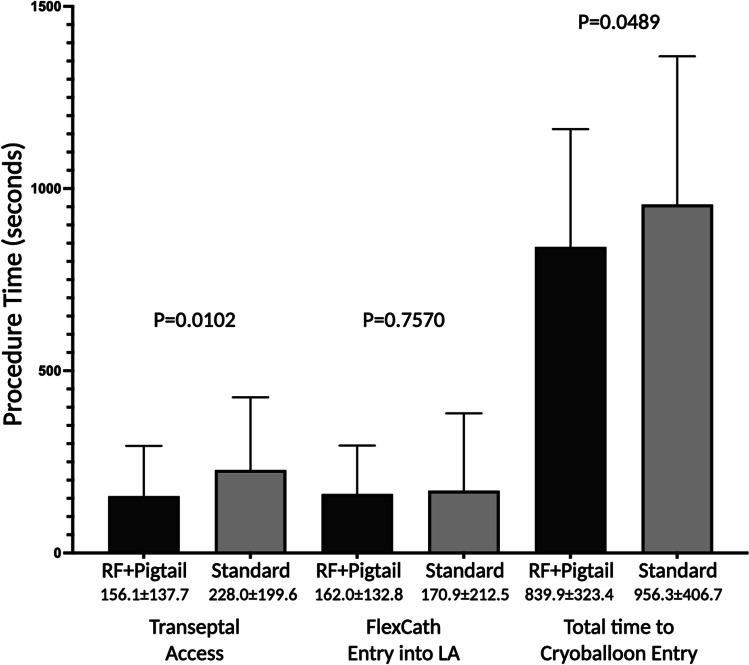
Transseptal puncture procedure time by assigned group. Plots show the mean and standard deviation. Total time to cryoballoon entry was defined as time from the first pull-down of the needle/sheath/dilator apparatus from the superior vena cava to first entrance of the cryoballoon catheter into the left atrium. Transseptal access was defined as the time from first pull-down to first entrance of the transseptal sheath into the left atrium. FlexCath entry to LA was defined as the time from engagement of the interatrial septum to FlexCath advancement into the left atrium. Times are inclusive of crossover time

The primary outcome of total time required for transseptal LA access as defined as time from the first pull-down of the needle/sheath/dilator apparatus in the superior vena cava to first entrance of the cryoballoon into the left atrium was significantly shorter in the RF + Pigtail group (839.9 ± 323.4 s) when compared to the standard group (956.3 ± 406.7 s, *P* = 0.0489, Fig. 2). The time from first pull-down to sheath advancement into the left atrium was significantly shorter in the RF + Pigtail group (156.1 ± 137.7 vs. 228.0 ± 199.6 s, *P* = 0.0102); however, the time from initial left atrial access to FlexCath sheath advancement was no different between groups (162.0 ± 132.8 vs. 170.9 ± 212.5 s, *P* = 0.7570).

### Fluoroscopy time

**Fig. 3:**
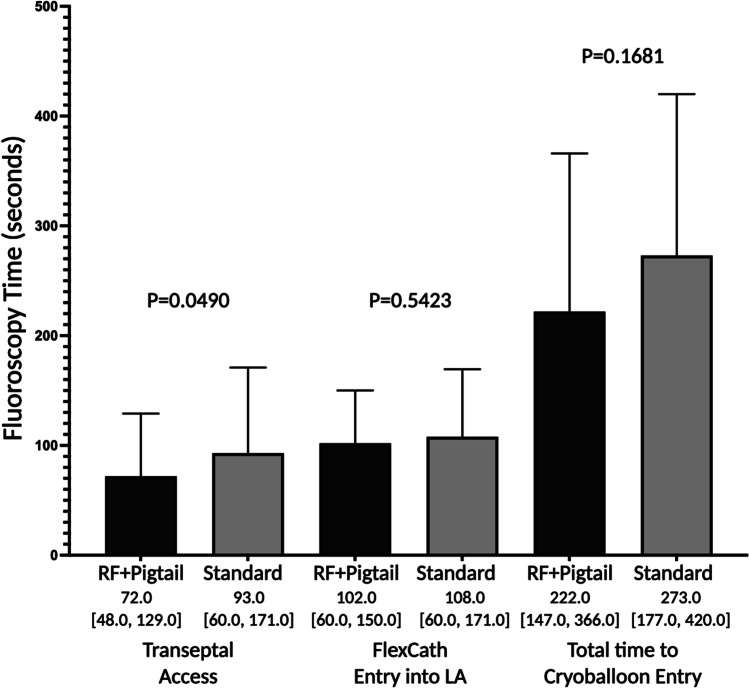
Fluoroscopy time by assigned group. Plots show the median with 95% confidence interval. Total time to cryoballoon entry was defined as time from the first pull-down of the needle/sheath/dilator apparatus from the superior vena cava to first entrance of the cryoballoon catheter into the left atrium. Transseptal access was defined as the time from first pull-down to first entrance of the transseptal sheath into the left atrium. FlexCath entry to LA was defined as the time from engagement of the interatrial septum to FlexCath advancement into the left atrium. Times are inclusive of crossover time

The total fluoroscopy time required for left atrial access was 222.0 s (IQR 147.0, 366.0) in the RF + Pigtail group vs. 273.0 s (IQR 177.0, 420.0) in the standard group, *P* = 0.1681 (Fig. 3). The fluoroscopy time required for left atrial access was significantly shorter in the RF + Pigtail group (72.0 [IQR 48.0, 129.0] vs. 93.0 [IQR 60.0, 171.0] seconds, *P* = 0.0490); however, the time from initial left atrial access to FlexCath sheath advancement was not significantly different between groups (102.0 [IQR 60.0, 150.0] vs. 108.0 [60.0, 171.0] seconds, *P* = 0.5423).

### Transseptal location

**Fig. 4:**
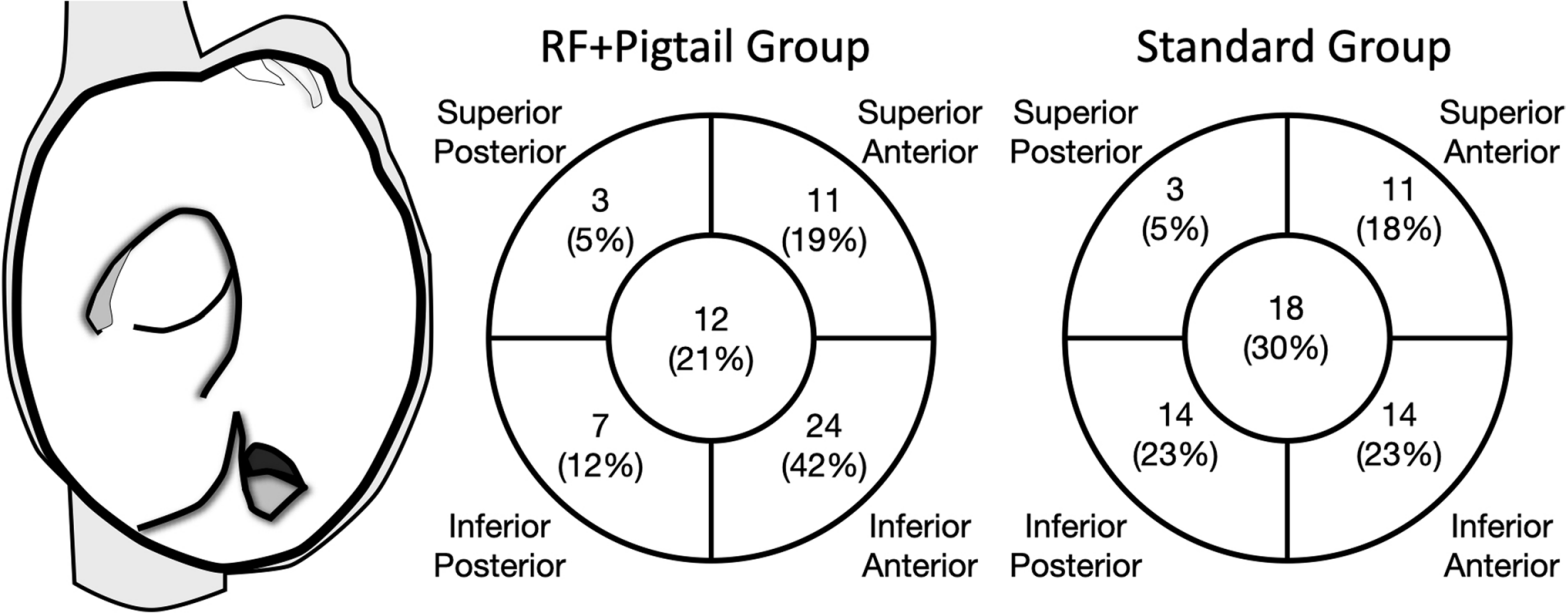
Transseptal puncture location. The distribution of transseptal puncture location for patients undergoing echocardiographically guided transseptal access. An inferior-anterior position is more desirable for cryoballoon ablation procedures due to difficulty engaging the right inferior pulmonary vein with the deflectable sheath

In those patients who underwent echocardiographically guided transseptal puncture, patients in the RF + Pigtail group were more often observed to have an ideal inferior-anterior transseptal location (42.1% vs. 23.3%, *P* = 0.0476, Fig. 4).

### Safety outcomes

No procedural complication related to transseptal puncture was observed in either group.

## Discussion

The CRYO-LATS trial is the first multicenter randomized comparison of transseptal access, comparing the radiofrequency needle/stiff pigtail transseptal system to conventional left atrial access tools. The trial observed that the radiofrequency needle/stiff pigtail transseptal system significantly lowered the procedural time to left atrial access and the fluoroscopy time used to attain left atrial access. This was likely influenced by a significant reduction in transseptal attempts and a trend towards a lower rate of failure to achieve left atrial access with the radiofrequency needle/stiff pigtail transseptal system.

Percutaneous access to the left heart via transseptal puncture was first described in the late 1950s as a diagnostic procedure [6]. Following early improvements in equipment and technique [7], the use of transseptal puncture waned following the advent of the pulmonary artery catheter in the 1970s [8]. However, recent years have seen a resurgence in transseptal puncture coincident with the expansion of catheter ablation and structural heart procedures necessitating access to the left heart.

The conventional transseptal puncture approach employs a fixed curve 18-gauge needle (e.g., 19° or 53°) introduced through a long sheath under fluoroscopic guidance. Although percutaneous transseptal access to the left atrium is usually achieved in a safe and dependable manner, there continues to be a significant incidence (1–2%) of major complications including tamponade, aortic puncture, or thromboembolism [9, 10]. Even for skilled operators, the procedure is technically demanding, necessitating a sound anatomical understanding given variations in the interatrial septum (size, thickness, elasticity, orientation, or aneurysm) or atria, with complex transseptal procedures increasing the risk of ionizing radiation exposure to the patient and operator, as well as prolonging procedure times.

More recently, there has been an evolution in the technologies employed for the treatment of AF and structural heart interventions (e.g., left atrial appendage occlusion and mitral valve intervention), which necessitate the passage of large bore sheaths and complex devices into the left atrium through the interatrial septum. While traditional transseptal tools remain effective, the use of these complex, large, left atrial catheters and devices has compelled renewed interest in developing safer and more effective transseptal equipment.

One such development is the NRG transseptal needle, which delivers radiofrequency energy through a blunt closed tip device. The main advantage of this device is the reduced need for excessive mechanical pressure, potentially reducing excessive “jump” and the subsequent complications of catheter exit into the pericardial space, as well as facilitating an easier crossing in cases of hypermobile, thickened, or scarred septa, with the latter being commonly observed during repeat left atrial procedures. Observational studies using the RF needle have reported shorter procedure time, shorter fluoroscopy time, and increased efficacy, [11, 12] findings that have been replicated in a single-center randomized controlled trial [13] as well as the current larger multicenter randomized controlled trial. Consistent with the previous studies, we observed a significant 30% reduction in the time to left atrial access, with more uniform time to left atrial access in the RF needle group, which was driven by significantly less puncture attempts, a lesser need for transseptal apparatus repositioning, and a lesser need to crossover from the assigned transseptal group. In addition, we observed a significant 20% reduction in fluoroscopy time required for left atrial access.

In contrast, while a small retrospective observational study using an older generation of the atraumatic stiff body pigtail wire for mitral valve intervention suggested that its use was associated with a significant reduction in the time to placement of the 22-French steerable guide catheter [14], we did not observe any significant difference in procedure or fluoroscopy time for placement of the 15-French steerable cryoballoon ablation sheath. In contrast to the previous study, we differentiated the time required for each step in the transseptal procedure, observing that the majority of the reduction in transseptal procedure time was attained with the NRG needle rather than the use of the pigtail wire. Moreover, our study was a randomized comparison, whereas the previous study was a sequential observational analysis, in which a learning curve effect may have accounted for their observed reduction in left atrial access time.

### Limitations

The current study exclusively enrolled patients undergoing a first ablation procedure. It is known that repeat transseptal punctures are more technically challenging as the septum becomes thick, scarred, or calcified [15]. It is possible that the observed difference between groups may have been greater if we had included patients with previous transseptal access, a population where the RF needle or atraumatic stiff pigtail wire may hold a theoretical advantage [13].

## Conclusions

The use of the radiofrequency needle/stiff pigtail transseptal system resulted in shorter time to left atrial access and reduced fluoroscopy time compared to left atrial access using conventional transseptal equipment in this multicenter randomized clinical trial of patients undergoing initial cryoballoon ablation.

## Abbreviations

AF: Atrial fibrillation
IQR: Interquartile range

## References

[1] Andrade J, Khairy P, Dobrev D, Nattel S (2014). The clinical profile and pathophysiology of atrial fibrillation: relationships among clinical features, epidemiology, and mechanisms. Circ Res.

[2] Andrade JG, Wazni OM, Kuniss M, Hawkins NM, Deyell MW, Chierchia GB, Nissen S, Verma A, Wells GA, Turgeon RD (2021). Cryoballoon ablation as initial treatment for atrial fibrillation: jacc state-of-the-art review. J Am Coll Cardiol.

[3] Andrade JG, Deyell MW, Badra M, Champagne J, Dubuc M, Leong-Sit P, Macle L, Novak P, Roux JF, Sapp J (2017). Randomised clinical trial of cryoballoon versus irrigated radio frequency catheter ablation for atrial fibrillation-the effect of double short versus standard exposure cryoablation duration during pulmonary vein isolation (CIRCA-DOSE): methods and rationale. BMJ Open.

[4] Andrade JG, Champagne J, Deyell MW, Essebag V, Lauck S, Morillo C, Sapp J, Skanes A, Theoret-Patrick P, Wells GA (2018). A randomized clinical trial of early invasive intervention for atrial fibrillation (EARLY-AF) - methods and rationale. Am Heart J.

[5] Andrade JG, Wells GA, Deyell MW, Bennett M, Essebag V, Champagne J, Roux JF, Yung D, Skanes A, Khaykin Y (2021). Cryoablation or drug therapy for initial treatment of atrial fibrillation. N Engl J Med..

[6] Ross J, Braunwald E, Morrow AG (1959). Transseptal left atrial puncture; new technique for the measurement of left atrial pressure in man. Am J Cardiol.

[7] Brockenbrough EC, Braunwald E, Ross J (1962). Transseptal left heart catheterization. A review of 450 studies and description of an improved technic. Circulation.

[8] Swan HJ, Ganz W, Forrester J, Marcus H, Diamond G, Chonette D (1970). Catheterization of the heart in man with use of a flow-directed balloon-tipped catheter. N Engl J Med.

[9] O'Brien B, Zafar H, De Freitas S, Sharif F (2017). Transseptal puncture - review of anatomy, techniques, complications and challenges. Int J Cardiol.

[10] De Ponti R, Cappato R, Curnis A, Della Bella P, Padeletti L, Raviele A, Santini M, Salerno-Uriarte JA (2006). Trans-septal catheterization in the electrophysiology laboratory: data from a multicenter survey spanning 12 years. J Am Coll Cardiol.

[11] Fromentin S, Sarrazin JF, Champagne J, Nault I, Philippon F, Molin F, Blier L, O'Hara G (2011). Prospective comparison between conventional transseptal puncture and transseptal needle puncture with radiofrequency energy. J Interv Card Electrophysiol.

[12] Winkle RA, Mead RH, Engel G, Patrawala RA (2011). The use of a radiofrequency needle improves the safety and efficacy of transseptal puncture for atrial fibrillation ablation. Heart Rhythm.

[13] Hsu JC, Badhwar N, Gerstenfeld EP, Lee RJ, Mandyam MC, Dewland TA, Imburgia KE, Hoffmayer KS, Vedantham V, Lee BK (2013). Randomized trial of conventional transseptal needle versus radiofrequency energy needle puncture for left atrial access (the TRAVERSE-LA study). J Am Heart Assoc.

[14] Buchner S, Dreher A, Resch M, Schach C, Birner C, Luchner A (2015). Simplified method for insertion of steerable guide into the left atrium using a pigtail guide wire during the MitraClip((R)) procedure: a technical tip. J Interv Cardiol.

[15] Marcus GM, Ren X, Tseng ZH, Badhwar N, Lee BK, Lee RJ, Foster E, Olgin JE (2007). Repeat transseptal catheterization after ablation for atrial fibrillation. J Cardiovasc Electrophysiol.

